# The association between dietary inflammatory index and C-reactive protein in plasma and semen with semen quality: A cross-sectional study

**DOI:** 10.18502/ijrm.v21i10.14539

**Published:** 2023-11-24

**Authors:** Ali Taheri Madah, Saeid Hadi, Beheshteh Abouhamzeh, Vahid Hadi, Maasoume Abdollahi, Kiumars Omidi

**Affiliations:** ^1^Department of Anatomical Science and Research Center, Aja University of Medical Sciences, Tehran, Iran.; ^2^Department of Health and Nutrition, School of Medicine, Aja University of Medical Sciences, Tehran, Iran.; ^3^Department of Radiology, School of Paramedicine, Shahid Beheshti University of Medical Sciences, Tehran, Iran.

**Keywords:** Infertility, C-reactive protein, CRP, Inflammation, Sperm.

## Abstract

**Background:**

Infertility affects couples worldwide, with male factors being responsible for half of all cases.

**Objective:**

This study aimed to investigate the relationship between dietary inflammatory index (DII) and levels of C-reactive protein (CRP) in plasma and semen with the quality of semen in infertile males.

**Materials and Methods:**

In this cross-sectional study, 88 infertile men referring to Besat hospital, Tehran, Iran from December 2021-November 2022 were enrolled. A detailed questionnaire requesting information, and a 168-item semiquantitative food frequency questionnaire, were completed by participants. A food frequency questionnaire was used to calculate the DII. Additionally, semen and blood samples were collected from each participant for semen analysis and CRP-level assessment. Statistical analyses were performed to explore the association between DII and CRP levels with sperm quality. The correlation between DII and serum/semen CRP, besides assessing nutrients in each DII quartile group, was also explored.

**Results:**

A significant difference was observed between different DII quartiles considering sperm motility (p = 0.006) and morphology (p = 0.014). Post hoc study revealed a significant difference between the 1 ${}^{\text{st}}$ and 2 ${}^{\text{nd}}$ quartiles and the 1 ${}^{\text{st}}$ and 4 ${}^{\text{th}}$ quartiles of DII regarding sperm motility (p = 0.011, and 0.017 respectively) and a significant difference between the 1 ${}^{\text{st}}$ and 2 ${}^{\text{nd}}$ quartiles of DII considering sperm morphology (p = 0.009). A statistically significant inverse correlation was also observed between DII and sperm motility (p *=* 0.017). Carbohydrates and β-carotenes were significantly different between the 4 DII quartiles (p = 0.043 and p = 0.026, respectively). Finally, no significant correlation was observed between DII and CRP levels in blood and semen (p > 0.05).

**Conclusion:**

The findings suggested a notable correlation between DII and semen quality; however, no significant association were observed between DII and CRP levels in blood and semen.

## 1. Introduction

Approximately 8-15% of couples struggle with infertility worldwide (1). In this instance, males individually comprise 20-30% of infertility cases and currently contribute to 50% of all cases (2, 3). Besides known hormonal disorders (4), other physical and physiological conditions may also play a role in male infertility, including environmental aspects like diet and toxins (5, 6), genetic disorders (7, 8), as well as inflammation and infection (9). However, low sperm numbers, poor sperm quality, or both are the reason in more than 90% of cases (4).

Pro-inflammatory cytokines in the male reproductive tract may serve some physiological functions; however, inflammation might also have toxic effects on spermatogenesis (9, 10). In this regard, inflammation in the male reproductive tract can lead to ejaculatory duct obstruction, epididymitis, and oxidative stress (OS) (11). OS, triggered by inflammation and various factors including tobacco use, alcohol consumption, obesity, leukocytospermia, and viral infections, has been found to be a significant contributor to sperm damage and male infertility (12). Increased production of reactive oxygen species and inadequate antioxidant capabilities in sperm might result in sperm DNA fragmentation, apoptosis, and impairment of sperm motility (11, 13).

Better semen quality is correlated with a healthy diet low in saturated fatty acids (SFA) and trans fatty acids and high in nutrients such omega-3 fatty acids, antioxidants, and vitamins (14, 15). Conversely, diets high in SFA and low in polyunsaturated fatty acids have been linked to reduced sperm quality (16). On the other hand, a high intake of alcohol, caffeine, red meat, and processed meat negatively influences fertility (14). Consumption of pro-inflammatory products, having a low antioxidant intake, and following a high glycemic index diet can promote OS (17, 18). In obese individuals, hypothalamic-pituitary-gonadal axis disorders and pro-inflammatory cytokines from adipose tissue can also cause systemic inflammation and OS (19). Leptin released by adipocytes may negatively affect testicular function in infertile males. However, studies show controversial results (6).

The liver manufactures C-reactive protein (CRP), an acute phase reactant, in response to interleukin (IL) 2 stimulation, notably IL-6 and it is used to assess the presence and severity of infectious and inflammatory diseases (20). In 2009, the dietary inflammatory index (DII) was created as a tool for categorizing people diets on a scale ranging from highly anti-inflammatory to maximally pro-inflammatory (21). A connection between inflammation and DII factors, particularly CRP, IL-6, and tumor necrosis factor-α (TNF-α), in adolescents and adults, indicating that diets with high DII scores are linked to increased inflammatory markers (22).

So far, no evidences have been found on the relationship between DII and male infertility that comprehensively assesses all foods for their inflammatory potential.

Therefore, this study aimed to investigate the association of DII and CRP levels in plasma and semen with the quality of semen in infertile men.

## 2. Materials and Methods

### Study population

This cross-sectional study was conducted on 88 men with primary infertility who sought evaluation and treatment at the infertility clinic of Besat hospital, Tehran, Iran from December 2021-November 2022. Eligible participants meeting the inclusion criteria underwent collection of semen and blood samples, and also completed a comprehensive questionnaire. The questionnaire covered various aspects including demographic information, socioeconomic status, lifestyle factors, medical history, and a dietary assessment. We established 2 inclusion criteria for male participants in our study: at least 1 yr of regular intercourse without prevention and failure in pregnancy and an age range of 20-40 yr. Conversely, we excluded men who had a history of any chronic diseases (such as diabetes, cancer, multiple sclerosis or other immunologic conditions, cardiovascular diseases, stroke, amnesia, thyroid and liver diseases, or varicocele), as well as those who consumed alcohol or tobacco.

### Semen and blood samples

Before sample collection, participants were told to avoid ejaculation for 3-5 days. Masturbation was used to gather samples of the sperm into polypropylene containers in the laboratory and incubated in a 37 C incubator for 15-30 min until complete liquefaction was achieved. Our trained colleagues conducted the semen analyses, which involved assessing semen volume, sperm concentration, total sperm count, proportion of immotile sperm, and percentage of increasing sperm motility. These parameters were evaluated in accordance with the guidelines set forth by the World Health Organization (23). CRP levels were also measured in the semen samples. Furthermore, a peripheral blood sample was collected from each patient after an overnight fast to assess the CRP levels in their blood plasma.

### General questionnaire

A general information questionnaire was completed by the researchers, including age, education status, socioeconomic status, smoking, history of medical diseases, and use of medications or supplements.

### Food frequency questionnaire (FFQ)

Data about dietary status was collected using a 168-item semiquantitative FFQ, of which the validity and reliability have already been confirmed (24). The researchers completed the questionnaires during interviews. The semiquantitative food consumption questionnaire includes 168 food items that shows the status of macronutrient and micronutrient intake during the past year. In this questionnaire, people were asked how many times a day, week, month, and year a specific share size of 168 food items was consumed.

### DII

Dietary information from the FFQ was used to calculate the DII. The DII was calculated using the average and global standard deviation (SD) of 45 various food characteristics, mostly flavonoids, micro- and macronutrients, and some specific food items. Based on the global database obtained from 11 countries. Z-score and middle percentile of each dietary parameter were calculated for each individual in the study. For this purpose, each food item was adjusted for energy by the residual method. Then, to calculate the z-score, these values were deducted from the average global standard reception and divided into global SD. To reduce asymmetry, the values came in percentile, and finally, the percentile values were multiplied by the inflammatory score of the desired food parameter. The inflammatory rating of each food item was calculated based on the previous study (21). The total inflammatory index of each individual's diet was obtained from the total DII score for the dietary parameter of each food. Based on 29 dietary parameters (instead of 45 dietary parameters), the present study was conducted to calculate the DII considering the lack of consumption of some food items in Iranian food culture and the lack of some items such as polyphenols in our food database.

### Measurement of serum CRP

The enzyme-linked immunosorbent assay method was used to measure the levels of serum CRP by using relevant laboratory kits. The cytokine enzyme was used to determine the serum CRP levels with the help of an enzyme-linked immunosorbent assay kit and immunosorbent assay. Specific antibodies and dyes were utilized in this method.

### Ethical considerations

The study was conducted following the latest revision of the Declaration of Helsinki. Before signing a written informed consent, a detailed description of the study was distributed to the eligible participants. The study protocol was approved by the Ethical Committee of Medical University for the Islamic Republic of Iran's Army, Tehran, Iran (Code: IR.AJAUMS.REC.1400.249).

### Statistical analysis

SPSS statistic 25 (International Business Machines corporation, Armonk, NY, USA) was used for statistical analysis. Continuous variables were reported as mean ± SD, and categorical variables were reported as frequency (percentage). Continuous variables were compared using the independent *t* test and one-way ANOVA, and categorical variables were compared using the Chi-square or Fisher's exact test. The correlation between variables was evaluated using the Pearson test. The relationships between the DII and pro-inflammatory factors of blood and semen plasma with semen parameters were assessed using linear or logistic regression statistical tests after modulating for confounding factors. P-value < 0.05 was considered statistically significant.

## 3. Results

### Subjects

At the outset, the study population consisted of 145 individuals; however, 57 male participants were subsequently excluded from the study due to noncompliance with inclusion and exclusion criteria. A total of 88 subjects were assessed, of which 41 subjects had normal semen analyses while 47 subjects had abnormal semen analyses. The mean age of participants with normal and abnormal semen analyses was 34.92 ± 5.29 and 35.36 ± 4.63, respectively. There was no significant difference in body mass index (BMI) between the groups (Table I).

### Sperm quality assessment

An analysis of variance (ANOVA)-based statistical analysis found a significant difference in sperm count, motility, and morphology between the normal and abnormal semen analyses groups (p < 0.001). However, no significant differences were observed in terms of semen volume and CRP levels in both semen and serum (Table I).

### DII

To evaluate the communication between DII and variables in table I, DII was divided into quartiles which refer to the division of the study population's DII scores into 4 equal groups (quartile I = -4.96 to -4.68; quartile II = -3.66 to -3.13; quartile III: -2.69 to -2.49; and quartile IV = -1.86 to -0.81). The 1 ${}^{\text{st}}$ quartile represents the group with the lowest DII scores, indicating a diet with higher anti-inflammatory potential. The 4 ${}^{\text{th}}$ quartile comprises the group with the highest (most positive) DII scores, suggesting a diet with a more pro-inflammatory profile. The 2 ${}^{\text{nd}}$ and 3 ${}^{\text{rd}}$ quartiles represent intermediate levels of inflammatory potential based on their respective DII scores. Significant differences were observed between different DII quartiles in motility (p = 0.006) and morphology variables (p = 0.014) (Table II). Further, significant differences were observed in motility between the 1 ${}^{\text{st}}$ and 2 ${}^{\text{nd}}$ quartiles of DII (p = 0.011, MD = 19.18) as well as the 1 ${}^{\text{st}}$ and 4 ${}^{\text{th}}$ quartiles (p = 0.017, MD = -18.31) (Table II).

In addition, a significant difference in sperm morphology was observed between the 1 ${}^{\text{st}}$ and 2 ${}^{\text{nd}}$ quartiles of DII. Age, BMI, sperm count, volume, and total motile count did not demonstrate significant differences among the different DII quartiles (p > 0.05) (Table II).

The Pearson correlation method was employed to assess the relationship between DII and semen analysis parameters. Notably, a significant inverse correlation was observed between DII and sperm motility. However, no significant correlations were found between DII and other semen analysis parameters, including sperm count, morphology, and semen volume. Moreover, no significant associations were observed between DII and CRP levels in both blood and semen (Table III).

A regression model was used to evaluate the effect of the DII quartile on semen analysis parameters. The analysis shows that the 2 ${}^{\text{nd}}$ quartile significantly increases the chances of abnormal semen analysis. However, the results for the 3 ${}^{\text{rd}}$ and 4 ${}^{\text{th}}$ quartiles were insignificant (p > 0.05). The same results were replicated when the analysis was adjusted for age and BMI of subjects for DII quartile II (Table IV).

The ANOVA test was used to evaluate nutritional variables in different DII quartiles (Table V), and a significant difference was observed between DII quartiles in carbohydrates and β-carotenes (p = 0.043 and p = 0.026, respectively). Using the pos hoc test, this significant difference was found between the 1 ${}^{\text{st}}$ and 4 ${}^{\text{th}}$ DII quartiles. Other nutritional variables did not exhibit significant differences across different DII quartiles (p > 0.05).

**Table 1:** Comparison of demographic and laboratory characteristics between normal semen analysis and abnormal semen analysis groups


**Variables**	**Normal analysis (n = 41)**	**Abnormal analysis (n = 47)**	**P-value**
**Age (yr)**	34.92 ± 5.29	35.36 ± 4.63	0.68
**BMI (kg/m^2^)**	27.28 ± 3.11	27.56 ± 4.07	0.72
**Sperm count**	24.48 ± 9.63	13.85 ± 8.82	< 0.001*
**Sperm motility (%)**	53.95 ± 18.00	30.31 ± 17.55	< 0.001*
**Sperm morphology (%)**	4.53 ± 0.80	2.48 ± 1.10	< 0.001*
**Semen volume (mL)**	2.59 ± 0.70	2.28 ± 0.81	0.06
**Serum CRP (ng/mL)**	2.73 ± 1.44	2.61 ± 1.30	0.67
**Semen CRP (ng/mL)**	2.28 ± 1.03	2.34 ± 0.97	0.76
Data was presented as Mean ± SD. ANOVA test. *Shows a significant difference (p < 0.05). BMI: Body mass index, CRP: C-reactive protein			

**Table 2:** Comparison of demographic and sperm characteristics among DII quartiles


	**DII quartiles**				
**Variables**	**I (n = 22)**	**II (n = 22)**	**III (n = 22)**	**IV (n = 22)**	**P-value**
	**Age (yr)**	35.41 ± 5.12	35.77 ± 5.10	34.23 ± 4.84		35.23 ± 4.86	0.76
**BMI (kg/m^2^)**	28.22 ± 4.30	26.31 ± 2.98	27.86 ± 3.27	27.35 ± 3.82	0.33
**Sperm count**	18.00 ± 11.37	15.91 ± 9.60	19.23 ± 9.78	22.09 ± 11.29	0.27
**Sperm motility (%)**	52.77 ± 24.16	33.59 ± 18.44	44.50 ± 19.74	34.45 ± 17.36	< 0.001*
**Sperm morphology (%)**	4.00 ± 1.11	2.68 ± 1.29	3.64 ± 1.33	3.45 ± 1.63	0.01*
**Semen volume (mL)**	2.41 ± 0.73	2.45 ± 0.86	2.68 ± 0.78	2.18 ± 0.68	0.20
**Total motile count (%)**	24.45 ± 20.32	14.64 ± 14.31	25.24 ± 22.12	20.90 ± 19.52	0.25
Data was presented as Mean ± SD. ANOVA test. *Shows a significant difference (p < 0.05). BMI: Body mass index, DII: Dietary inflammatory index					

**Table 3:** The correlation between DII and other study variables


**Variables**	**Pearson correlation coefficient (r)**	**P-value**
**Sperm count**	0.159	0.13
**Sperm motility**	-0.254	0.01*
**Sperm morphology**	-0.070	0.51
**Semen volume**	-0.063	0.55
**Total motile count**	-0.005	0.96
**CRP (serum)**	-0.097	0.37
**CRP (semen)**	0.081	0.45
The Pearson correlation method. *Shows a significant difference (p < 0.05). CRP: C-reactive protein, DII: Dietary inflammatory index		

**Table 4:** Adjusted and unadjusted regression models assessing the role of DII scores in the development of abnormal semen analysis


**Variables**	**Odds ratio**	**95% Confidence interval**	**P-value**
**Unadjusted values**			
**DII quartile II**	5.95	1.58-22.32	< 0.001*
**DII quartile III**	1.75	0.52-5.84	0.36
**DII quartile IV**	1.75	0.52-5.84	0.36
**Adjusted values**			
**DII quartile II**	6.738	1.727-26.293	< 0.001*
**DII quartile III**	1.830	0.539-6.209	0.33
**DII quartile IV**	1.830	0.548-6.306	0.31
**Age**	1.013	0.925-1.110	0.78
**BMI**	1.060	0.938-1.199	0.35
*Shows a significant difference (p < 0.05). DII: Dietary inflammatory index, BMI: Body mass index			

**Table 5:** Assessment of nutrients in each group of DII quartiles


	**DII quartiles**				
**Variables**	**I (n = 22)**	**II (n = 22)**	**III (n = 22)**	**IV (n = 22)**	**P-value**
	**Energy (Kcal/day)**	2736.54 ± 1043.48	2841.4268 ± 969.00	3146.1495 ± 1237.42		3491.2845 ± 1050.17	0.09
**Protein (g/day)**	97.48 ± 38.64	99.12 ± 31.16	121.77 ± 85.47	118.01 ± 51.12	0.34
**Carbohydrate** **(g/day)**	399.37 ± 122.38	413.48 ± 168.46	448.35 ± 147.59	519.62 ± 155.64	0.04*
**Fat (g/day)**	83.23 ± 52.84	87.88 ± 34.39	96.18 ± 50.35	104.53 ± 42.59	0.42
**SFA (g/day)**	26.25 ± 19.53	26.40 ± 10.59	29.70 ± 16.22	33.80 ± 20.67	0.40
**MUFA (g/day)**	23.23 ± 16.69	25.24 ± 11.79	27.19 ± 17.30	29.14 ± 12.65	0.42
**PUFA (g/day)**	17.14 ± 10.40	18.91 ± 10.08	19.90 ± 12.41	19.59 ± 6.72	0.58
**Magnesium (mg/day)**	350.44 ± 153.29	321.73 ± 134.39	330.59 ± 169.56	303.55 ± 132.04	0.80
**Fiber (g/day)**	25.24 ± 11.26	22.86 ± 10.58	20.73 ± 10.62	21.03 ± 7.64	0.43
**Zinc (mg/day)**	10.5184 ± 6.02	11.19 ± 4.77	12.00 ± 9.04	11.49 ± 8.47	0.92
**Selenium (mg/day)**	0.0529 ± 0.03	0.0560 ± 0.044	0.0469 ± 0.03	0.0434 ± 0.02	0.62
**Vit-A (IU/day)**	2183.00 ± 1979.43	1605.59 ± 1679.09 (IQR: 1488.9)	1482.37 ± 1002.86	1128.60 ± 604.07	0.10
**B-carotene (mg/day)**	1555.78 ± 1780.09	937.11 ± 567.42	670.028 ± 500.52	481.94 ± 325.97	0.02*
**Vit-E (IU/day)**	4.74 ± 2.12	4.66 ± 1.91	4.45 ± 1.73	4.84 ± 1.49	0.91
**Vit-B1 (mg/day)**	2.18 ± 0.74	2.21 ± 0.64	2.50 ± 0.91	2.71 ± 0.75	0.07
**Vit-B2 (mg/day)**	1.84 ± 0.98	1.59 ± 0.55	1.98 ± 1.16	1.80 ± 1.25	0.65
**Vit-B3 (mg/day)**	25.35 ± 9.70	26.53 ± 7.69	31.98 ± 19.42	29.42 ± 7.73	0.27
**Vit-B6 (mg/day)**	1.95 ± 1.00	1.86 ± 0.90	1.93 ± 1.53	1.59 ± 0.67	0.66
**Folate (mg/day)**	442.81 ± 248.59	387.38 ± 226.36	341.65 ± 145.25	332.34 ± 130.59	0.22
**Vit-B12 (mg/day)**	4.73 ± 3.54	4.07 ± 1.89	6.36 ± 7.54 (IQR: 6.686)	3.71 ± 3.08	0.22
**Vit-C (mg/day)**	229.72 ± 119.95	186.16 ± 102.16	202.31 ± 186.38	169.34 ± 99.44	0.47
**Vit-D (IU/day)**	2.31 ± 1.16	1.48 ± 1.13	1.56 ± 1.70 (IQR: 1.51)	2.21 ± 2.63 (IQR: 2.34)	0.28
Data were presented as Mean ± SD. ANOVA test. *Shows a significant difference (p < 0.05). DII: Dietary inflammatory index, SFA: Saturated fatty acids, MUFA: Monounsaturated fatty acids, PUFA: Polyunsaturated fatty acids, IQR: Interquartile range					

## 4. Discussion

Our findings support the idea that a healthy dietary pattern is linked to higher semen quality. According to our findings, there is a considerable difference between different DII quartiles considering sperm motility and morphology. This significant difference was observed between the 1 ${}^{\text{st}}$ and 2 ${}^{\text{nd}}$ quartiles, as well as the 1 ${}^{\text{st}}$ and 4 ${}^{\text{th}}$ quartiles of DII, regarding sperm motility. Moreover, a significant difference was observed in sperm morphology between the 1 ${}^{\text{st}}$ and 2 ${}^{\text{nd}}$ quartiles of DII. The significant association between DII and abnormal semen analysis was also seen when the analysis was adjusted for age and BMI. Carbohydrates and β-carotenes were the 2 nutrients significantly different between the 1 ${}^{\text{st}}$ and 4 ${}^{\text{th}}$ quartiles of DII. Finally, no correlation was observed between DII and CRP levels in blood and semen.

Inflammation is a crucial factor in male infertility, and higher seminal plasma concentrations of IL-1, IL-6, interferon-γ, and TNF-α have been found in infertile patients compared to normal controls (25). Moreover, TNF-α is capable of inducing sperm apoptosis (26). On the other hand, some studies have shown that nutrition can reduce inflammation. The Mediterranean diet is the most efficient at reducing inflammation. However, dietary approaches to arrest hypertension and a plant-based dietary model have been shown to be beneficial in reducing IL-6 and CRP levels (27). A recent meta-analysis revealed that adopting a healthy dietary pattern is associated with significant reductions in CRP (weighted mean difference, -0.75 [-1.16, -0.35]; p = 0.0003), although no other biomarkers were affected. The sub-group analysis demonstrated that this effect was observed in studies involving a Mediterranean diet and an intervention period of 3 months or more (28).

Limited studies assess the association between DII and inflammatory indices in plasma and semen with semen quality in men. A cross-sectional study of 209 young healthy male students in southern Spain found that an anti-inflammatory diet could be associated with increased sperm count. However, it does not necessarily affect sperm count, morphology, or reproductive hormones in young men. In this study, DII was significantly associated with progressive sperm motility (p = 0.03) and total sperm motility (p = 0.04) (29). Other dietary factors and their association with semen quality have been studied, as well. It was shown that high levels of dietary iron intake might be associated with reduced sperm concentration (p = 0.01) and the percentage of progressively motile sperm (p = 0.004) (30). Dietary methods for controlling hypertension have been demonstrated to enhance sperm concentration, total sperm count, and total motile sperm count in the young, healthy male population (31). However, no studies have been conducted on infertile men. To our knowledge, this is the 1 ${}^{\text{st}}$ study focusing on the association between DII and inflammatory indices with semen quality in the infertile male population. In an observational cross-sectional observe of 219 younger guys withinside the Western Australian Pregnancy Cohort (Rain) observe, sperm awareness and dihydrotestosterone-3α-diol have been negatively correlated with a “Western” nutritional pattern (p = 0.007 and; p = 0.044, respectively) and serum estradiol concentrations have been undoubtedly correlated with a “Western” nutritional pattern (p = 0.007) (32). Similar results were demonstrated in a study of 2935 Danish participants (5). Nonetheless, many controversial studies show positive, null, and negative relationships between a western or healthy diet and sperm quality (15, 33, 34).

A cross-sectional study of 7282 young male Taiwanese subjects demonstrated that high intake of a “Western diet” resulted in statistically linear declines in sperm concentration and normal sperm morphology. Furthermore, consuming snacks high in sugar and sweetened beverages, along with diets rich in carbohydrates and sodium, were correlated with lower sperm concentration, sperm motility, and normal sperm morphology, respectively (35). A systematic review of observational studies shows that a healthy diet rich in nutrients such as omega-3 fatty acids, some antioxidants, and vitamins and low in SFA and trans fatty acids is associated with improved semen quality parameters. Moreover, diets wealthy in fish, shellfish and seafood, poultry, cereals, greens and fruits, low-fats dairy, and skimmed milk had been substantially related to improved sperm quality. Conversely, processed meat, soy foods, potatoes, full-fat dairy and total dairy products, cheese, coffee, alcohol, sugar-sweetened beverages, and sweets have been associated with lower-quality of semen (14). A large clinical trial of 336 men attending an infertility clinic for diagnosis found a positive association between careful eating habits (including high amounts of fish, chicken, fruit, cruciferous vegetables, tomatoes, green leafy vegetables, legumes, and whole foods) and entire grains and sperm concentration and testosterone levels (p = 0.05, p = 0.03, respectively). In contrast, no association was observed with a “Western” dietary pattern (15).

This study has some weaknesses that need to be addressed, because they can limit the interpretation of the results. 1 ${}^{\text{st}}$ , we used questionnaires that rely on retrospective retrieval of information from memory which can be compromised as time passes. 2 ${}^{\text{nd}}$ , our study was a small, single-center, observational study that consisted of a small number of participants, and overgeneralization of findings to larger populations might not be feasible. 3 ${}^{\text{rd}}$ , both nutritional assessment and time since last ejaculation were self-reported, and our population may be exposed to environmental factors that may impact the observed associations. Finally, potential variables that may affect the reported relationships include health status, medication use, physical activity, energy consumption, and abstinence time.

## 5. Conclusion

Our study shows that there might be a significant association between DII and semen quality (specifically, sperm motility and morphology). However, we found no correlation between DII and CRP levels in blood and semen. Considering the limitations of our study, additional studies with more participants and prospective designs are needed to show the causation of these associations and their effects on sperm quality.

##  Conflict of Interest

The authors declare that there is no conflict of interest.
